# Memristors with Monolayer Graphene Electrodes Grown Directly on Sapphire Wafers

**DOI:** 10.1021/acsaelm.4c01208

**Published:** 2024-09-16

**Authors:** Zhichao Weng, Robert Wallis, Bryan Wingfield, Paul Evans, Piotr Baginski, Jaspreet Kainth, Andrey E. Nikolaenko, Lok Yi Lee, Joanna Baginska, William P. Gillin, Ivor Guiney, Colin J. Humphreys, Oliver Fenwick

**Affiliations:** † School of Physical and Chemical Sciences, Queen Mary University of London, London E1 4NS, United Kingdom; ‡ School of Engineering and Materials Science, Queen Mary University of London, London E1 4NS, United Kingdom; § Paragraf Limited, 7-8 West Newlands, Somersham PE28 3EB, Cambridgeshire, United Kingdom

**Keywords:** graphene, memristor, wafer-scale, MOCVD, transfer-free, metal-catalyst-free

## Abstract

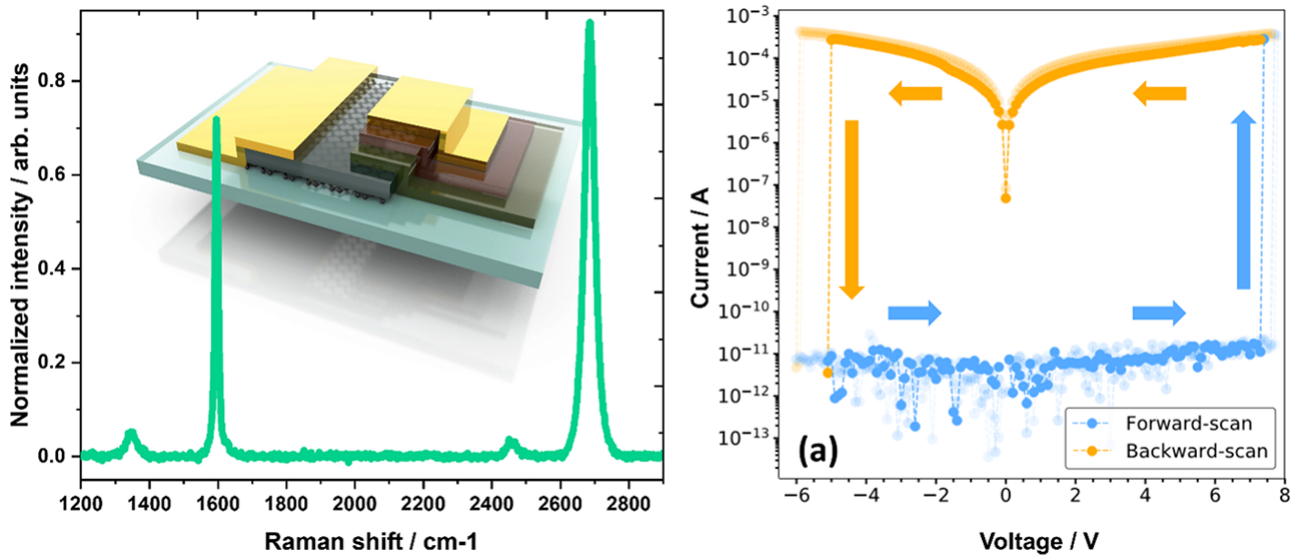

The development of the memristor has generated significant interest due to its non-volatility, simple structure, and low power consumption. Memristors based on graphene offer atomic monolayer thickness, flexibility, and uniformity and have attracted attention as a promising alternative to contemporary field-effect transistor (FET) technology in applications such as logic and memory devices, achieving higher integration density and lower power consumption. The use of graphene as electrodes in memristors could also increase robustness against degradation mechanisms, including oxygen vacancy diffusion to the electrode and unwanted metal ion diffusion. However, to realize this technological transformation, it is necessary to establish a scalable, robust, and cost-effective device fabrication process. Here we report the direct growth of high-quality monolayer graphene on sapphire wafers in a mass-producible, contamination-free, and transfer-free manner, using a commercially available metal–organic chemical vapor deposition (MOCVD) system. By taking advantage of this approach, graphene-electrode based memristors are developed, and all the processes used in the device fabrication incorporating graphene electrodes can be performed at wafer scale. The graphene electrode-based memristor demonstrates promising characteristics in terms of endurance, retention, and ON/OFF ratio. This work presents a possible and viable route to achieving robust graphene-based memristors in a commercially and technologically sustainable manner, paving the way for the realization of more powerful and compact integrated graphene electronics in the future.

## Introduction

The demand for computing power and data storage is growing rapidly, requiring a fast evolution of the global semiconductor industry,^1,2^ so it is important to explore new types of semiconductor devices based on technology other than conventional FETs (field-effect transistors). In computing technologies, higher scalability and integration density, lower power consumption, faster processing speed, lower manufacturing cost, and sustainability are all important factors to meet this demand,^3,4^ and the memristor is one of the most promising candidates to achieve this.

The memristor is a unique electronic device, having an internal resistance which can be switched ON or OFF and an internal state which is dependent on its previous operational history. This unique type of switching behavior differs from conventional semiconductor devices (e.g., FETs) and offers a new type of computing device. The theory of memristive behavior was established around 50 years ago.^5^ However, it was only in 2008 that they were experimentally realized, leading to significant attention from industry.^4,6^ Memristors have certain advantages, for example, non-volatility, a simpler manufacturing process and lower power consumption, over some conventional semiconductor devices.^7^

Memristors have considerable potential in various applications, such as logic circuits, RRAM (resistive random-access memory),^8^ and neuromorphic computing,^9−11^ etc. In particular, the memristor structure of electrode/insulator/electrode promises a significantly simpler fabrication process compared to FET-based technologies, which could reduce the fabrication cost and time substantially compared to FGMOS (Floating-gate MOSFET) based flash memory, for example. Recently, scientists have been incorporating 2D materials in memristor structures, offering a route to a higher integration density as well as lower power consumption and higher performance.^12^

Graphene is a promising electrode material in memristors due to its flexibility, high transparency, high conductivity, high uniformity and single-atom thickness.^13−15^ Graphene is chemically quite stable, and this will feed into the endurance of graphene electrode based devices.^16^ It has also been found that graphene memristors also do not show significant compromise between device flexibility and performance robustness.^17^ Graphene, as the electrode(s) in memristor devices, could also bring down the manufacturing cost significantly, as an effective alternative to conventional but expensive noble metal electrodes (e.g., Au, Pt, etc.) used in memristors.^18−20^ However, one of the most important advantages of using graphene electrodes in memristors is the improvement of the robustness of the device and reduction of device degradation. In cation memristors using metal filament formation/elimination as the switching mechanism, uncontrolled metal cation diffusion is a major degradation mechanism. In anion memristors, oxygen vacancy diffusion forms conductive filaments, and transparent conductive oxide electrodes are often used, in particular where transparency is useful (e.g., in optoelectronic memristor devices). In this case degradation can occur by oxygen vacancy diffusion across the electrode-dielectric interface in the region of the filament.^21^ The covalent in-plane sp^2^ hybridized bonding of graphene is substantially stronger than metal–metal bonding (∼700 kJ mol^–1^ and ∼200 kJ mol^–1^, respectively), and it does not support oxygen vacancy diffusion, making it stable against these degradation mechanisms. Furthermore, the use of graphene electrodes has great potential for photonic memristors due to its high transparency and sustainability compared to the commonly used counterpart, indium tin oxide (ITO),^22−24^ noting that Indium is one of nine endangered elements listed by the American Chemical Society.^25^ However, it is still challenging to produce a large-area monolayer graphene on technologically relevant substrates in a commercially effective manner.

Large area graphene synthesis has been reported using different deposition methods, for example chemical vapor deposition (CVD)^26,27^ of monolayer graphene on metal foils, and solution-based processes for the deposition of thin films of graphene flakes,^28^ making it possible to produce low-cost but high density and flexible graphene electronic components. CVD graphene grown on metal foils can be of high quality, but a transfer step onto a relevant substrate for semiconductor devices is then required, and this transfer step is hard to scale commercially. Films made from graphene flakes do not achieve the same performance in terms of charge mobility or transparency as monolayer graphene.

Here, we report the fabrication of memristors incorporating monolayer graphene electrodes directly grown on sapphire substrates without any metal catalyst using a commercially available metal–organic chemical vapor deposition (MOCVD) system (AIXTRON CRIUS MOCVD) in batches of up to 37 wafers. The whole procedure is compatible with standard equipment and processes of the semiconductor industry, such as photolithography, MOCVD, thermal evaporation, and atomic layer deposition (ALD). This ensures that our graphene-based device fabrication is both cost-effective and contamination-free, enabling the scalability and industrialization of graphene-based electronics. The as-grown graphene is shown to be of high quality and monolayer by Raman spectroscopy. Following photolithography and device fabrication, the patterned graphene electrode retains its high quality, evident from Raman spectroscopy. Our memristors show promising performance in low voltage switching (*V*_set_ = 1.60 V and *V*_reset_ = −1.55 V), stability (switching operation remains stable and robust after 2730 cycles) and ON/OFF ratios of up to >10^7^, even in their prototype form. These results are comparable to memristors either with more complex device structures,^29,30^ more expensive materials (e.g., Au for both electrodes^31−33^) or made with toxic elements (e.g., MoSe_2_, MoS_2_^34,35^), and the devices are optically accessible through the transparent graphene electrode.

## Results and Discussion

**Figure 1:**
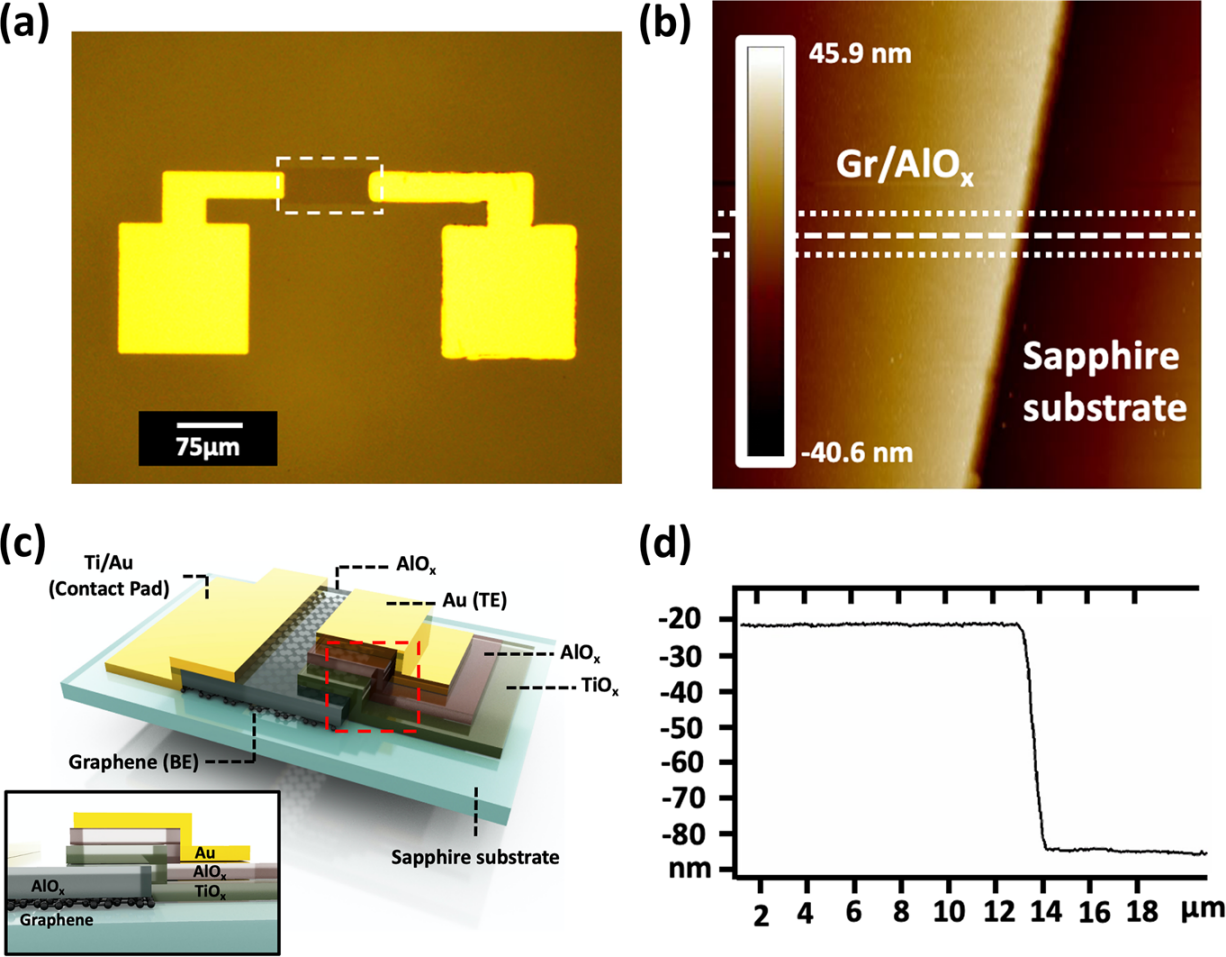
(a) Microscopic view of the graphene-electrode memristor. Inside the dashed area is the patterned graphene/AlO_*x*_ strip. (b) AFM image of the edge of a patterned graphene/AlO_*x*_ strip. (d) Line profile across the section shown as the dashed line. (c) 3D configuration of our graphene-electrode memristor. The memristive junction is highlighted in the dashed square area with the inset illustrating the details of the junction (Au - AlO_*x*_ - TiO_*x*_ - Graphene). In this device structure, Au is used for the top electrode (TE) and graphene is used as the bottom electrode (BE).

**Figure 2:**
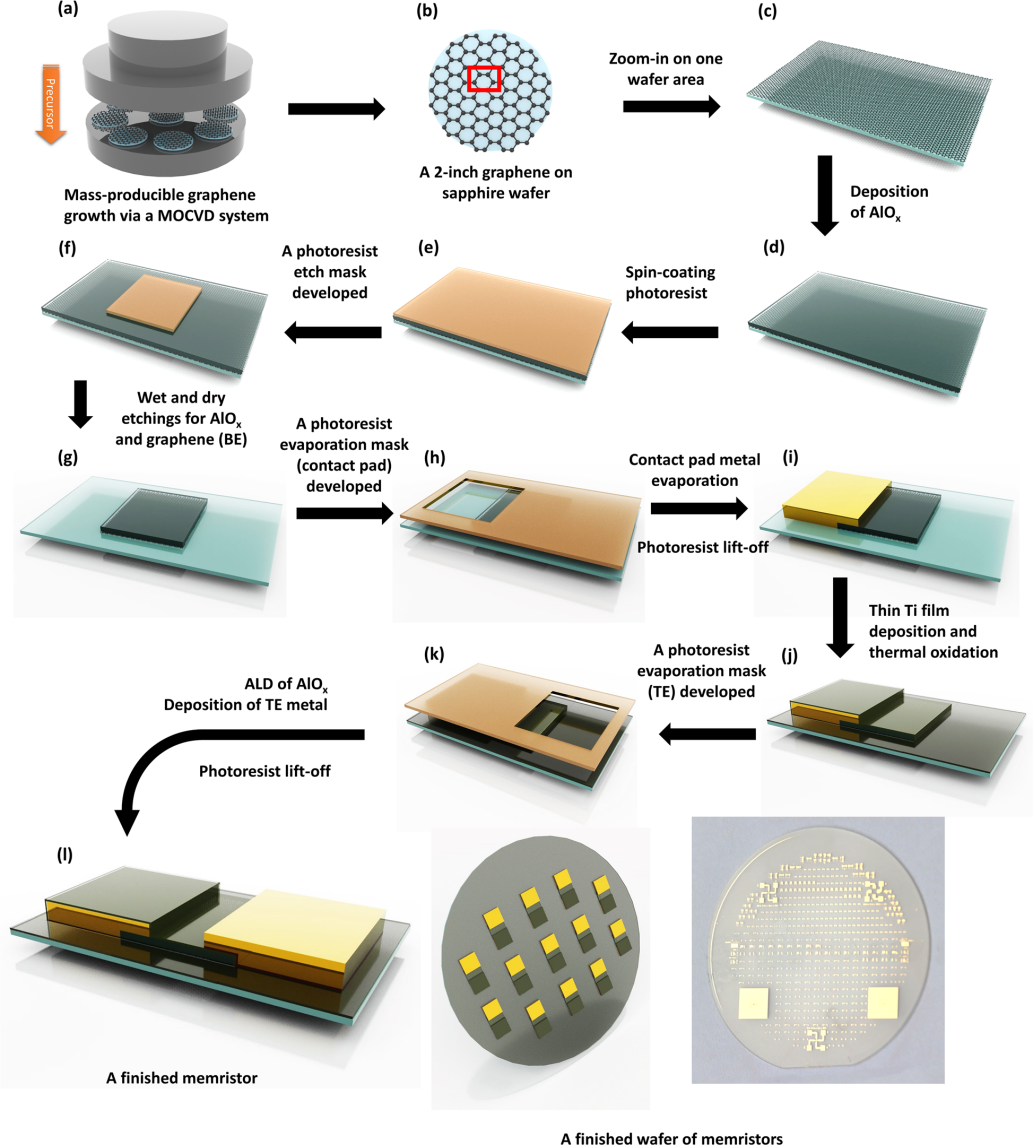
General process flow for the graphene-electrode based memristor fabrication. (a) Large-scale monolayer graphene on sapphire wafers growth via an industry standard MOCVD system; (b) one graphene wafer from a single fabrication batch and (c) is the zoomed-in area on the wafer. (d) A thin layer of AlO_*x*_ is thermally evaporated on top of the wafer. (e–g) Graphene bottom electrode (BE) definition. Photoresist is spin-coated on top of the wafer, and a photoresist etch mask is developed through an industry standard photolithography process. Wet and dry etchings are performed on AlO_*x*_ and graphene separately for the patterning of the active area. (h) and (i) Contact pad definition. A photolithography process is performed to develop a contact pad evaporation mask, followed by metal (Ti/Au) deposition and resist/residual metal lift-off process. (j–l) Memristor active materials deposition and lift-off. Deposition of Ti thin film following by thermal oxidations for TiO_*x*_ formation (repeated once to increase thickness). Then another photolithography process is performed for defining the area for dielectric and TE. This is followed by a thin AlO_*x*_ layer deposition via atomic layer deposition (ALD) and metal deposition by thermal evaporation (Au). A final step of resist/residual metal lift-off is performed before the device finish-up. The completed wafer of memristor devices is illustrated through both a schematic diagram and photograph (Copyright © Paragraf Limited 2024) at the end.

**Figure 3:**
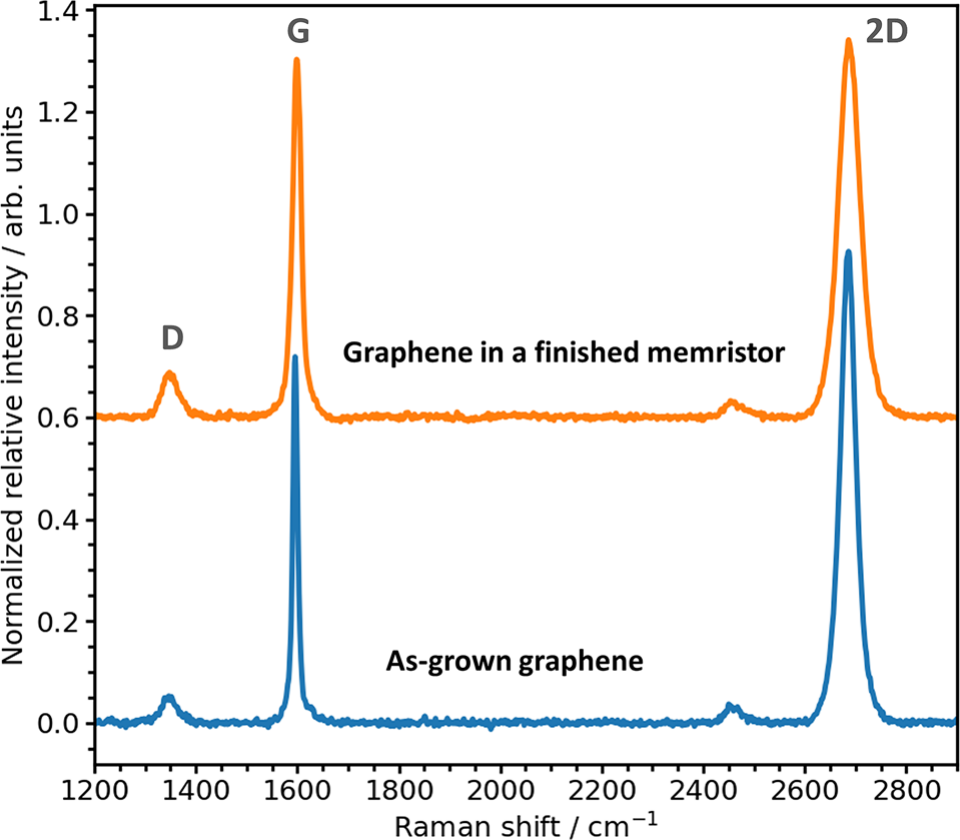
Raman spectra (excitation at 532 nm) after background subtraction of the as-grown graphene on a sapphire substrate and graphene in a finished memristor after several steps of photolithography. The calculated peak area ratio characteristics are *I*_D_/*I*_G_ = 0.25 ± 0.02 and *I*_2D_/*I*_G_ = 3.96 ± 0.16 for as-grown graphene; *I*_D_/*I*_G_ = 0.25 ± 0.04 and *I*_2D_/*I*_G_ = 2.26 ± 0.21 for graphene in a finished memristor. The fwhm of the G peak are 11.8 ± 0.7 for the as-grown graphene and 15.0 ± 1.6 for the graphene in a finished memristor. The mean values are the arithmetic average of the data for 3–4 different points across 2 different wafers, and the error bars are the standard errors that resulted from the error propagation of both the experimental errors and the spectra fitting errors.

Figure 1 shows our wafer and device configurations. Our graphene was directly grown on 2-in. sapphire substrates in a commercially available metal–organic chemical vapor deposition (MOCVD) system (AIXTRON CRIUS MOCVD) in a batch of 37 wafers by an established method.^36^ MOCVD systems were originally applied to III–V compound semiconductor production using TMGa-AsH_3_, TEIn-PH_3_, TEIn-AsH_3_–PH_3_ and other precursors.^37^ However, materials other than the metalorganic precursors can also be used for synthesis,^38,39^ allowing it to act as a conventional chemical vapor deposition system (CVD). The general device fabrication process, illustrated in Figure 2, is industry-compatible and performed in air in a clean room. This includes 4 rounds of photolithography (an extra step of alignment marks development is not shown in Figure 2); 4 rounds of lift-off processes at 80 °C for 2 h each; photoresist stripping, wet etching, high temperature Ti layer oxidation (200 °C for 1 h in air two times), and plasma etching. Although graphene is known to be sensitive to external stimuli (e.g., chemicals,^40^ air,^41^ water,^42^ etc.), the combination of our device configuration^43,44^ and the careful selection of nondestructive and noncontaminating industry-compatible fabrication processes ensured that the monolayer graphene survived these processes as indicated by the Raman spectra analysis (Figure 3).

Figure 1(a) and (c) illustrate our device configuration from both the microscopic view and 3D schematics. A thick AlO_*x*_ layer was thermally evaporated on top of the graphene sheet. This provides protection of the graphene and ensures that the active region of the memristor is in edge-contact with graphene. This novel edge-contact configuration^43,44^ provides a viable pathway for depositing a thin atomic layer deposited (ALD) dielectric layer as the active region of the memristor, resolving the difficulty in growing thin ALD layers directly on graphene surface due to lack of nucleation sites on the surface.^45^ For example, in the application of growing ultrathin tunnelling layers on top of the graphene surface,^45^ without any surface treatment (e.g., O_3_ plasma treatment), it is difficult to form a uniform and continuous thin ALD layer. Figure 1(b) displays an atomic force microscope (AFM) image of a patterned graphene/AlO_*x*_ strip prior to any further device processing. It shows a clean and sharp definition of the edge of the graphene/AlO_*x*_ structure, suggesting the effectiveness of our wet etching technique in the device processing. As shown in Figure 1(c), the graphene bottom electrode (BE), which is protected by an evaporated AlO_*x*_ layer, is connected to a contact pad (10 nm Ti/200 nm Au, left-hand side in Figures 1(a) and (c). The memristive junction (MJ) is formed at the other end of the graphene sheet by the inclusion of thin layers of TiO_*x*_ (thermal oxidation of 4 nm thermally evaporated Ti thin film) and AlO_*x*_ (atomic layer deposited at 4.5 nm) to form a thin dielectric between the graphene edge and the Au electrode.

In Raman spectroscopy analysis, the as-grown graphene on the sapphire substrate has an integrated intensity ratio *I*_2D_/*I*_G_ of 3.96 ± 0.16, which is within the typical range of graphene grown on sapphire.^46−49^ This larger intensity of the 2D band relative to the G band is a key characteristic of monolayer graphene. This larger relative intensity of the 2D band results from a triple resonance process, which is distinctive for sp^2^-hybridized carbon atoms.^50^ Additionally, a *I*_D_/*I*_G_ ratio of 0.25 ± 0.02 indicates a low defect density in the as-grown graphene.^51^ Further improvement in graphene defect density has been recently demonstrated at Paragraf Limited, as outlined in Figure S4 and analyzed in Table S4 in the Supporting Information. The characteristic frequencies of the D, G and 2D peaks are (1348.1 ± 1.3) cm^–1^, (1592.2 ± 1.6) cm^–1^, and (2685.3 ± 0.4) cm^–1^, respectively. Moreover, the shape of the 2D band in the Raman spectrum is also a distinctive feature for identifying single-layer graphene. As the number of graphene layers increases, the 2D band begins to split and exhibit asymmetry. Specifically, the 2D band in the Raman spectrum of monolayer graphene is uniquely symmetrical and can be accurately fitted with a single Lorentzian peak.^50,52^ Analysis of the Raman spectrum of our as-grown graphene, shown in Figure 3 and detailed (Figure S3) in the Supporting Information, reveals a highly symmetrical 2D peak that is fitted well with a single Lorentzian peak (fitting score *R*^2^ = 0.99685), confirming the as-grown graphene to be a monolayer. Notably, the patterned graphene in the device’s active area maintained the same low defect density (*I*_D_/*I*_G_ = 0.25 ± 0.04), even after several rounds of intense physical and chemical processes, indicating that the combination of our novel device structure design^36−38^ and our device fabrication process is nondestructive to graphene, even though graphene is known to be sensitive to external stimuli. The D peak frequency remained the same (1347.2 ± 1.1) cm^–1^, while the G peak frequency blueshifts to (1597.1 ± 0.9) cm^–1^ and 2D peak frequency redshifts to (2682.7 ± 1.6) cm^–1^ after the device fabrication. These shifts may be caused by many factors, for example, graphene doping during device processing, or external strain from the sapphire substrate, or the interplay between these two.^53^ A smaller value of *I*_2D_/*I*_G_ (2.3 ± 0.2) is also observed after device fabrication. It is known that the value of *I*_2D_/*I*_G_ indicates the level of interaction between graphene and its substrate, in our case, the sapphire substrate. The higher the value, the lower degree of interaction there is.^54^ Therefore, a reduction of the *I*_2D_/*I*_G_ value is expected after the device fabrication since there are multiple layers of material grown on top of the graphene. This is further demonstrated by the increased Raman peak widths and fwhm_G_ for the graphene in a finished memristor (fwhm_2D_ = (43.28 ± 2.83) cm^–1^, fwhm_G_ = (15.0 ± 1.6) cm^–1^) compared to that of the as-grown graphene (fwhm_2D_ = (36.89 ± 0.75) cm^–1^, fwhm_G_ = (11.8 ± 0.7) cm^–1^) as the 2D and G peaks widths broaden with the external out-of-plane compression effect on graphene surface.^55^ High-quality exfoliated single-layer graphene on sapphire also exhibits a comparably low fwhm_G_ = (12 ± 1) cm^–1^.^56^

**Figure 4:**
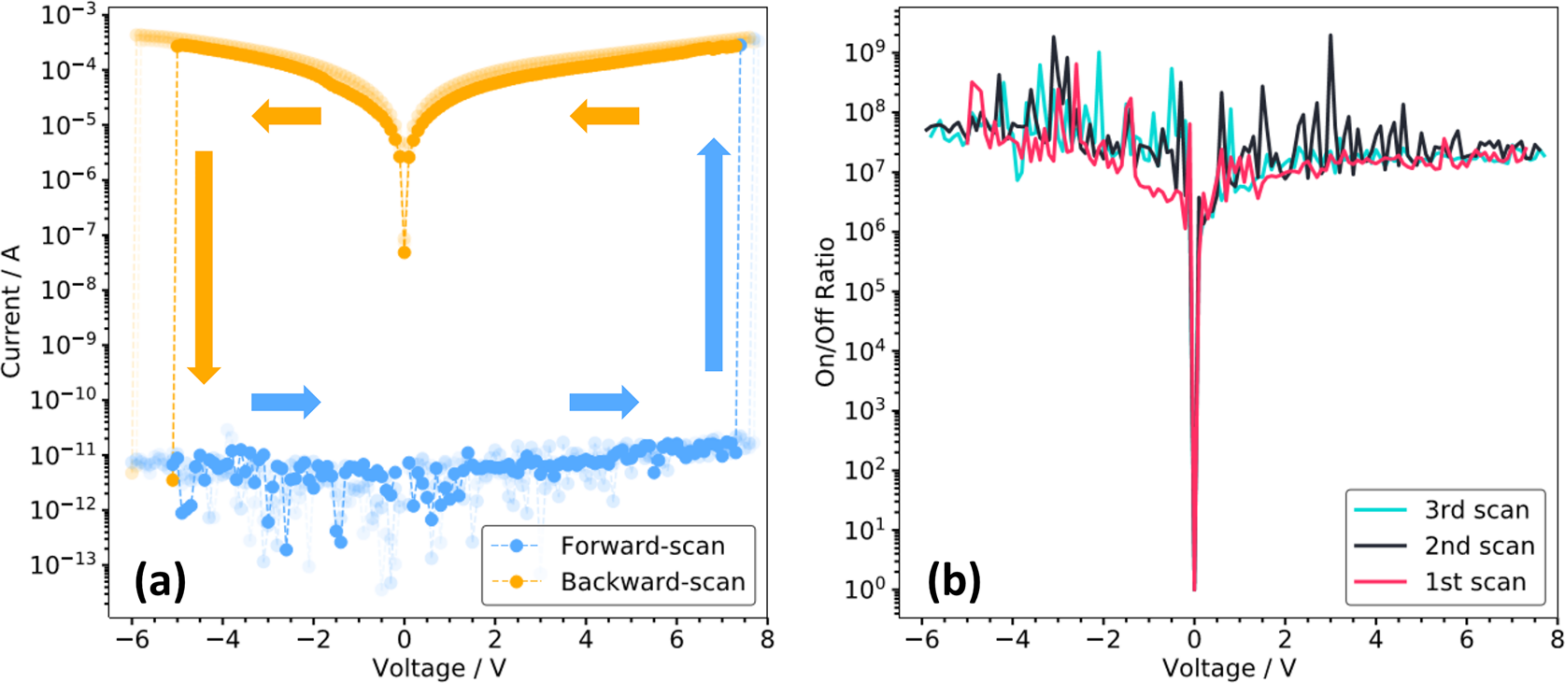
(a) Current–voltage (*IV*) initial scans of a single graphene-electrode memristor with a low sourcing rate (0.5 Hz–1 Hz). There are 3 rounds of consecutive scans with the first-round scan solid-color filling and the others slightly transparent for a better viewing purpose. The arrow indicates the voltage sweeping direction. (b) ON/OFF ratios calculated using the corresponding ON and OFF currents from (a) throughout the whole sourcing voltage range.

The current–voltage (*IV*) characteristics in Figure 4 were carried out using customized software with measurements starting at 0.1 V and increasing in steps of 0.1 V. In our electrical characterization, we sweep the voltage on the graphene electrode while keeping the Au electrode electrically grounded.The device current (*I*_d_) at each voltage step was measured and compared against the forward threshold device current (*I*_thf_, which was set as 10^–8^A). The forward scan was programmed to stop immediately after *I*_d_ exceeded *I*_thf_ (at this point, the device is considered as “switched on” due to the abrupt change of the device current). The backward scan immediately follows the forward one in steps of −0.1 V from the last voltage value where the device was switched on (*V*_set_). Likewise, *I*_d_ was continuously monitored and checked against the backward threshold device current (*I*_thb_, with |*I*_thb_| set as 10^–9^ A). Once |*I*_d_| fell below |*I*_thb_|, the backward scan process finished, and the forward scan took the voltage back to 0 V. The backward threshold voltage was registered as *V*_reset_. The voltage sourcing rate was in the range 0.5 Hz–1 Hz. When the device was fabricated, the IV scan was directly measured, as shown in Figure 4(a). Three consecutive scans were performed on the same device with the first scan result shown in solid-filled color and subsequent ones in partially transparent colors. The *V*_set_ are 7.4 V, 7.7 and 7.8 V, and *V*_reset_ are −5.1 V, −6 V and −5.9 V, for the corresponding 3 scans. The small variation in *V*_set_ (standard deviation (std) = 0.17) and *V*_reset_ (std = 0.40) is common in memristors due to the stochastic nature of conduction channel formation.^57,58^Figure 4(b) shows the calculated current ON/OFF ratio based on the values from Figure 4(a). Over the voltage range of −6 to 8 V, except for 0 V, the average ON/OFF ratio of the graphene-memristor lies between 10^7^ and 10^8^. Despite a simple two-layer structure and minimal optimsation, our device’ ON/OFF ratio of [10^7^, 10^8^] still rests in the midhigh value range in the memristor research community,^59,60^ and among those are state-of-the-art memristor devices with more complex structure designs and/or sophisticated memristive junction material combination selections. This shows the great potential for graphene electrode based memristors to achieve even higher ON/OFF ratios if further optimized, for example, by modifying the dielectric composition and thicknesses, etc. Furthermore, cross-wafer and cross-device evaluations were carried out on an additional 36 distinct graphene-electrode memristors (Figure S1 and analysis in Table S1). The statistical analysis underscores the reproducibility observed in our device performances and scalable fabrication processes.

**Figure 5:**
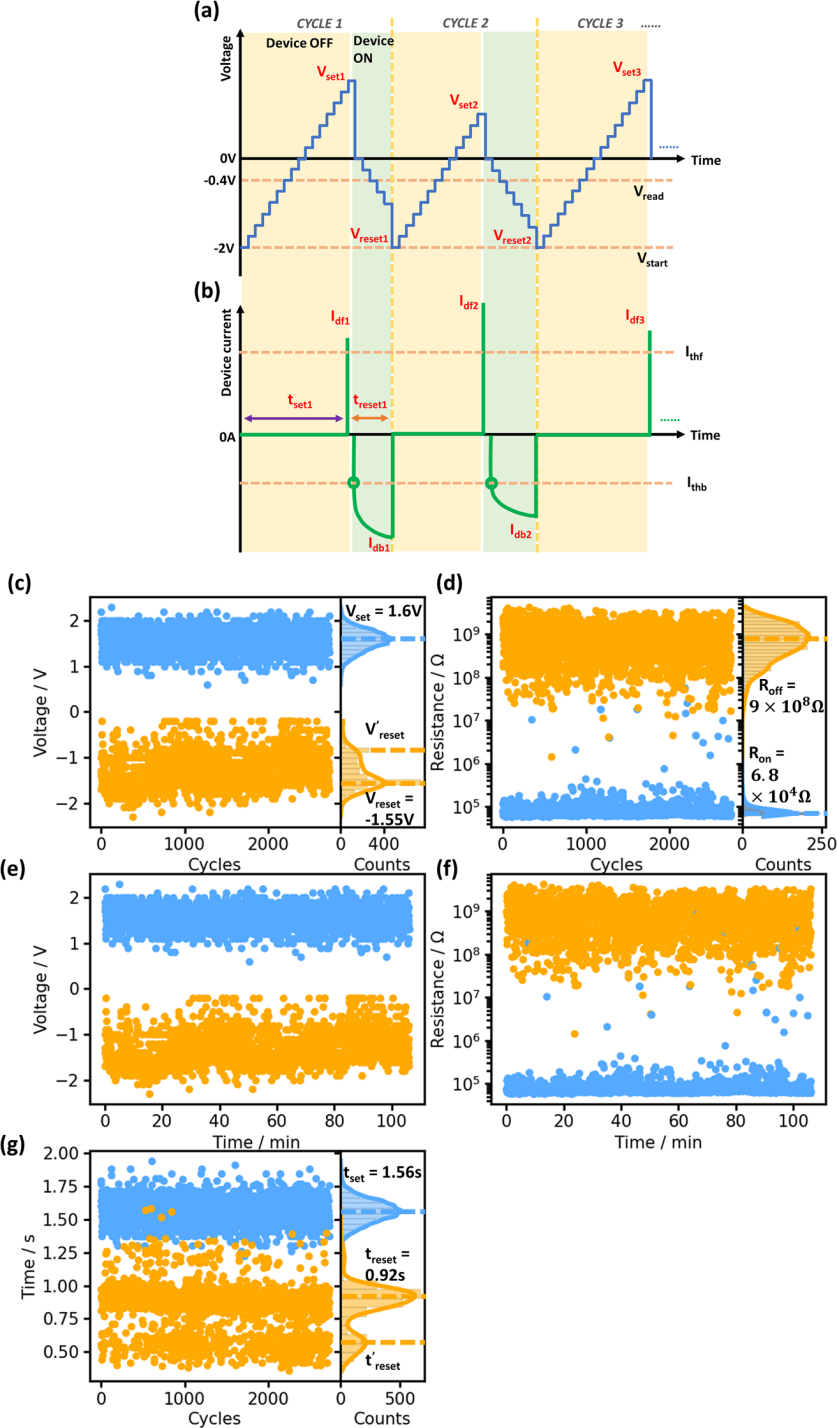
Endurance and retention characteristics of the graphene electrode based memristors. (a) and (b) Illustrations of memristor driving voltage signal patterns and device current. (c) and (d) Endurance characteristics and (e) and (f) retention characteristics for the device voltage (*V*_set_ and *V*_reset_) and the device resistance (*R*_on_ and *R*_off_), respectively. (g) Set and reset times over cycles.

The endurance and retention characteristics of our graphene-electrode memristor devices are shown in Figure 5. The device exists in OFF and ON states at different points in the endurance characterization cycle, as shown in Figure 5(a) and (b) as the yellow area and green area, respectively. In a single cycle, the driving voltage was initiated at −2 V (Figure 5(a)) and then scanned forward in increments of 0.1 V and a stepping rate of 26 Hz–46 Hz. Simultaneously, the forward device current, *I*_df_, was recorded and compared to a global forward threshold current, *I*_thf_, set in the software as shown in Figure 5(b). Once *I*_df_ exceeds *I*_thf_ (*I*_thf_ was set as 10 μA in our experiments), the memristor switches from the OFF state to the ON state, and the forward scan in cycle 1 is terminated by the software. A backward scan was programmed to follow immediately, starting from 0 V with a step of −0.1 V. Similarly, the backward device current, *I*_db_, was continuously monitored against a global backward threshold current, *I*_thb_ (notably, the device current at 0 V is explicitly excluded in this consideration to avoid a false registry of the reset voltage, *V*_reset_). Once the |*I*_db_| falls below |*I*_thb_| (|*I*_thb_| was set as 0.5 μA in these measurements), the memristor switches from the ON state back to the OFF state (shown in Figure 5(b)) and cycle 1 is finished. The last backward scan driving voltage is registered as *V*_reset_. The next cycle follows immediately. Additionally, during both the forward and backward scans, all the driving voltages and device currents are recorded by the software, and the device resistances (*R*_on_ and *R*_off_) are calculated using the recorded device currents (*I*_df_ and *I*_db_) at the reading voltage, *V*_read_, of −0.4 V as shown in Figure 5(a) within each cycle. The software also records the time durations for each scan, so they are used for retention checks as shown in Figure 5(e) and (f).

As shown in Figure 5(c)–(g), 2730 cycles of device switching *ON/OFF* performance are recorded over a period of 106 min. There is no sign of device degradation during the characterization with the set voltage, *V*_set_, and set resistance, *R*_set_, remaining constant, demonstrating promising stability in our graphene-electrode based memristor. Kernel Density Estimation (KDE) smoothing was used to produce histograms of *V*_set_, *V*_reset_, *R*_on_, and *R*_off_ (Figure 5(c) and (d)). The statistical *V*_set_ and *V*_reset_ were 1.60 V and −1.55 V respectively, which is comparable to the operational voltages of some state-of-the-art memristor devices^58,60^ and helps to keep the power consumption low. A secondary *V*′_reset_ peak (−0.87 V) appears as a shoulder next to the primary *V*_reset_ peak at −1.55 V. Additionally, from Figure 5(d), the *R*_on_ and *R*_off_ are statistically analyzed (i.e., the peak value of the histogram) to be 9.0 × 10^8^ Ω and 6.8 × 10^4^ Ω, respectively, yielding a statistical ON/OFF ratio of 1.3 × 10^4^. Figure 5(g) shows the evolutions of *t*_set_ and *t*_reset_ over the measurement cycles. There’s no sign of significant change of *t*_set_ and *t*_reset_, confirming the robustness and stableness of our device during the testing. Notably, there’s a clear secondary peak *t*′_reset_, in relevance to *V*′_reset_, and this is indicating that there could be two possible switch-off pathways within our graphene-based memristor with the slightly more resistive route as dominant (where *V*_reset_ = −1.55 V and *t*_reset_ = 0.92 s).^57^ Additionally, retention characterizations were carried out on 10 distinct graphene-electrode memristors from several batches of wafers, as presented in the Supporting Information, with statistical analysis indicating the reproducibility of the devices. The data depicted in Figure 5 is representative of the multiwafer data set (Figure S2 and analysis in Table S2).

## Conclusions

In conclusion, we have successfully fabricated a memristor featuring a monolayer graphene electrode directly deposited onto sapphire substrates using a commercially available MOCVD system capable of processing batches of up to 37 wafers. All processes were conducted on 2 in. sapphire wafers using industry-compatible methods. This includes the mass-producible, contaminant-free, and transfer-free growth of graphene, with device fabrication achieved entirely through vapor deposition and photolithography. The photolithography, etching, and lift-off processes were proven to be nondestructive to graphene by Raman characterization. The as-fabricated graphene-electrode memristors showed high ON/OFF ratios of 10^7^–10^8^ when under a bias stepping rate of 0.5 Hz–1 Hz. Notably, our graphene-electrode memristor demonstrated overall robust characteristics in endurance (switching remained stable after 2730 cycles), operational ON/OFF ratio (∼1.32 × 10^4^ under a bias pulsing rate of 26 Hz–46 Hz), stability (no device degradation after 1.8 h of switching at 26 Hz–46 Hz) and low voltage operation (low *V*_set_ and *V*_reset_ as 1.6 V and −1.55 V, respectively). Cross-device and cross-wafer characterizations and the statistical analysis highlight the robustness and reproducibility (see the Supporting Information) of device performance and fabrication processes. Our graphene electrode based memristor, even in its prototype form, shows competitive performance which is aligned with those displayed by some state-of-the-art memristor devices (an overview of recent studies on graphene-based memristor is demonstrated in Table S3), while eliminating the possibility of degradation due to unintended cation or vacancy diffusion from the electrode.^7,58−61^ Although graphene promises excellent electronic characteristics, the integration of graphene into commercial electronic devices has always been challenging due to the absence of a large-area and transfer-free fabrication technique for graphene. This work demonstrates a promising route to address this predicament by using an industry standard MOCVD system to directly deposit graphene onto a substrate, facilitating the development for more powerful electronics in computing industry and delivering a viable path toward the eventual realization of highly integrated graphene electronics.

## Methods

### Graphene Growth

Monolayer graphene was directly grown onto 2-in. sapphire substrates using a commercial MOCVD system (AIXTRON CRIUS MOCVD) by Paragraf Limited., and the detail can be found in ref.^62^ Using the current MOCVD model, up to 37 graphene on sapphire wafers can be produced in a single batch with the possibility of upscaling by using a larger capacity reactor model.

### Graphene-Electrode Memristor Processing and Fabrication

The graphene on sapphire wafers were directly obtained from a single graphene growth batch in the MOCVD system. Four customized photolithography masks (*M-I, M-II, M-III, M-IV*) were designed for the definitions of alignment marks (*M-I*), graphene/AlO_*x*_ area (*M-II*), Ti/Au electrodes (*M-III*), and Au electrodes (*M-IV*). 10 nm AlO_*x*_ layer was thermally evaporated on the whole wafer at a rate of 3 Å/s (MBRAUN Provap 5G deposition system), followed by the first photolithography process (*M-I*)/metal deposition/lift-off for the definition of alignment marks. A bilayer photoresist configuration (LOR7B lift-off resist and MICROPOSIT^TM^ S1813 photoresist) and AZ developer was used in the photolithography process. Then the graphene/AlO_*x*_ strip was patterned using the second photolithography process, combined with the wet etching of AlO_*x*_ using MICROPOSIT^TM^ 351 developer as the etching solution and the dry etching of graphene using oxygen plasma. The dry etching of graphene was performed using a Henniker Plasma Vacuum system (HPT-100) with 100% power and oxygen flow rate of 50 sccm for 8 min. A third photolithography process and 10 nm Ti/200 nm Au deposition/lift-off was carried out for the patterning of the electrode (*M-III*). Then the memristive junction was formed by the cycled deposition (2 cycles) of a 0.8 nm Ti film with a thermal oxidation process on a hot plate at 200 °C after each cycle. The thermal oxidation process was performed by exposing the sample to the air while incrementally increasing the hot plate temperature from room temperature (25 °C) to 200 °C. Once the hot plate temperature stabilized at 200 °C, the sample was maintained at this temperature for 40 min before being cooled down to room temperature. A photoresist mask for the top electrodes was then developed with a fourth photolithography process (*M-IV*) followed by the second memristive layer (4.5 nm AlO_*x*_) deposition through Atomic Layer Deposition (AT410 Anric Technologies). Lastly, a 200 nm layer of Au was thermally evaporated on top of the ALD AlO_*x*_ and a final lift-off was carried out to finish off the top electrode definition.

### AlO_*x*_ ALD Growth

The AlO_*x*_ thin film within the memristive junction was synthesized using a commercial ALD system (Anric Technologies, model: AT410). The ALD process involved the sequential introduction of precursor gases, specifically trimethylaluminum (TMA) and water. Prior to deposition, a 15 min prepurging phase was implemented to establish a stable and uniform growth environment. Subsequently, 40 cycles of thin film deposition were carried out, each consisting of 0.6 s pulses of TMA and 0.6 s pulses of water. The precursor flow rate was maintained at 27 sccm throughout the deposition process. The resulting thin film, growing at an approximate rate of 0.12 nm per cycle, was deposited over a total duration of approximately 50 min.

### Raman Spectroscopy

The Raman spectra were obtained by using a Thermo Scientific DXR Raman Microscope. A diode-pumped solid-state laser with a wavelength of 532 nm was used for the excitation with a grating of 1800 gr/mm. The measurement power was set as 5 mW with a 30 s acquisition time for each measurement under a ×100 objective lens. The Raman spectra for both the as-grown graphene and graphene in a finished device were obtained from different regions on different samples, and all the data displayed in the spectra were averaged across 3–4 samples for the purpose of spectra noise reduction and reproducibility check. The error bars displayed are standard errors propagated from a combination of the experimental error and data fitting error.

### Atomic Force Microscopy (AFM)

The AFM image for the characterization of the patterned graphene/AlO_*x*_ strip edge shown in Figure 1 was taken using a Bruker Dimension Edge AFM system in tapping mode. A SCOUT 350 RAI tip from Nu Nano Ltd. with a nominal radius of 5 nm was used. The AFM data was obtained and analyzed using the WSxM^63^ software program.

### Graphene-Electrode Memristor Electrical Characterizations

The measurement system comprises a probe station (EverBeing C-4), a source-measure unit (Keithley 2450) and a PC with the central control software, which was customized for the memristor characterization using Python.

## Supporting Information Available

Statistical analysis of graphene-electrode-based memristor performance across multiple wafers, the overview of graphene memristor performance in recent studies, and Raman spectrum analysis of as-grown graphene, including the most recent growth batch (PDF)
