# Supplementary material for: Memristors with Monolayer Graphene Electrodes Grown Directly on Sapphire Wafers
Source: ACS Appl Electron Mater. 2024 Sep 16;6(10):7276–85. doi: 10.1021/acsaelm.4c01208 (PMC11500406; doi:10.1021/acsaelm.4c01208)
Supplement: Supplementary file 1 — el4c01208_si_001.pdf [file el4c01208_si_001.pdf]

Supporting Information

Memristors with monolayer graphene electrodes grown directly on sapphire wafers

Zhichao Weng[1], Robert Wallis[3], Bryan Wingfield[3], Paul Evans[3], Piotr Baginski[3], Jaspreet Kainth[3], Andrey E. Nikolaenko[3], Lok Yi Lee[3], Joanna Baginska[3], William P. Gillin[1], Ivor Guiney[3], Colin J. Humphreys[2], Oliver Fenwick[2]**

[1] School of Physical and Chemical Sciences, Queen Mary University of London, London E1 4NS, United Kingdom

[2] School of Engineering and Materials Science, Queen Mary University of London, London E1 4NS, United Kingdom

[3] Paragraf Limited, 7-8 West Newlands, Somersham, Cambridgeshire, PE28 3EB, United Kingdom

1. Statistical Information on Graphene-Electrode based Memristors Performances

The current-voltage (IV) characteristics of a single memristor from a produced device wafer are illustrated in **Figure 4** of the main manuscript. To assess the reproducibility of our devices, IV characterizations were conducted on 36 different devices with varying sizes across multiple device wafers manufactured at different times over a 12-month period. The results are depicted in **Figure S1**, with all devices sharing the same structural configuration as demonstrated in **Figure 1**.

Statistical analysis of the collected data, as presented in **Figure S1 (b)** and **(c)**, reveals compelling insights into the consistency and reproducibility of our devices. Notably, the IV characteristics displayed in **Figure 4(a)** are represented within **Figure S1** as black triangle marked data points, illustrating their alignment with the broader dataset. This alignment is further supported by the statistical analysis showcased in **Figure S1 (b)** and **(c)**. **Table S1**

provides an overview of the extracted statistical information concerning ON/OFF Ratio, V_{set} and V_{reset}. The statistical analyses and calculations in **Figure S1** and **Table S1** exclude the data from the main text.

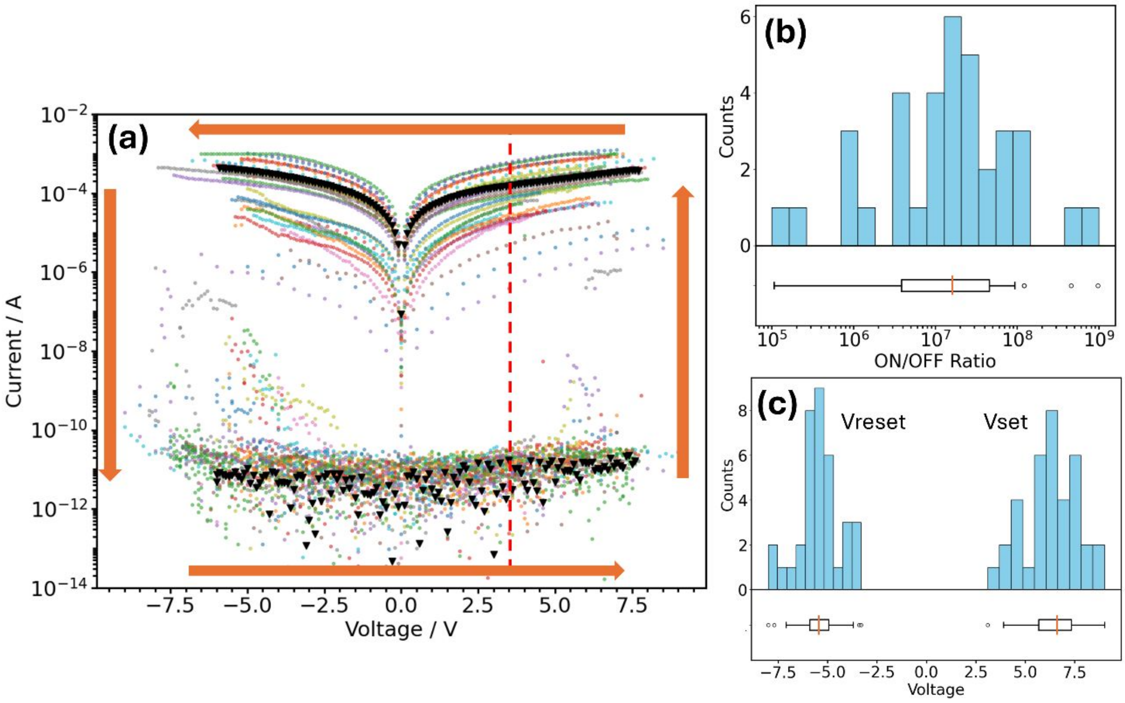

Figure S1. (a) IV scans of 36 distinct memristor devices across different wafers. The black triangles indicate data from **Figure 4(a)** for visual comparison. The orange arrow denotes the voltage sweeping sequence starting from device OFF state at certain negative voltage values. Histograms and boxplots of (b) ON/OFF ratio calculated at 3V and (c) V_{set} and V_{reset} distributions.

In our statistical analysis, the lower and higher whiskers defined in the boxplots (**Figure S1 (b-c)**) are important for understanding the range and variability of our memristor data. The lower whisker, extending from the edge of the box to the smallest observed data value within 1.5 times the interquartile range (IQR) below the first quartile (Q1), represents the lower bound of the "typical" range of the dataset. It indicates the minimum value within this range and serves as a threshold for identifying potential outliers. Conversely, the higher whisker extends from the edge of the box to the largest observed data value within 1.5 times the IQR above the third quartile (Q3). It represents the upper bound of the "typical" range of the data and indicates the maximum non-outlier value observed.

Table S1. Extracted statistical information regarding ON/OFF Ratio, V_{set} and V_{reset}.

	ON/OFF Ratio	V_{set} / V	V_{reset} / V

Mean	6.5×10^7	6.3	-5.4
Median	1.6×10^7	6.6	-5.5
Interquartile Range (IQR)	4.2×10^7	1.6	0.9
First Quartile (Q1 / 25% percentile)	3.9×10^6	5.7	-5.9
Third Quartile (Q3 / 75% percentile)	4.5×10^7	7.3	-4.9
Lower Whisker	1.1×10^5	3.9	-7.1
Higher Whisker	9.3×10^7	9	-3.7

In **Figure 4** of the main manuscript, $V_{set} = (7.6 \pm 0.17)$ V, $V_{reset} = (-4.7 \pm 0.4)$ V and the ON/OFF ratio is in the range 10^7 - 10^8. Those data points reside between the lower and the higher whiskers of all testing data, indicating that the data presented is indicative of performances across different devices and wafers.

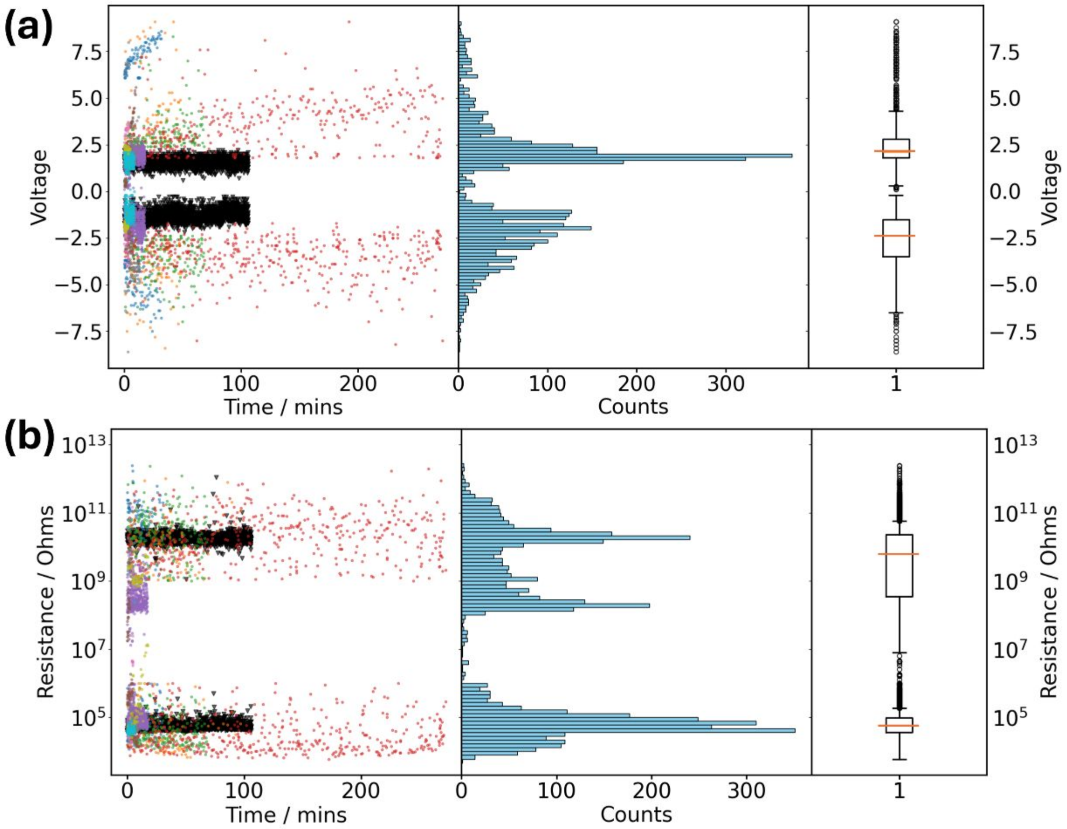

Figure S2. Retention characteristics for 10 distinct graphene-electrode based memristors from multiple wafers (a) switching voltages and (b) ON/OFF resistances with statistical analysis of histograms and boxplots of the data. The black triangles in both graphs indicate data reproduced from **Figure 5** in the main manuscript.

Moreover, a separate batch of 10 devices, distinct from those tested in **Figures S1**, **Figures 4**, and **Figures 5**, across several wafers underwent measurement for retention characteristics. The characterization results are depicted in **Figure S2** and statistically analyzed in **Table S2**. The statistical data from **Figure 5** are: V_{set} = 1.60 V, V_{reset} = -1.55 V, $R_{off} = 9.0 \times 10^8\ \Omega$ and $R_{on} = 6.8 \times 10^4\ \Omega$. Those values also lie well within the range between the lower whisker and the higher whisker in the statistical analysis in **Table S2**, also underscoring that data presented in the main manuscript is representative.

Table S2 Extracted statistical information regarding R_{on}, R_{off}, V_{set} and V_{reset}.

	V_{set} / V	V_{reset} / V	R_{on} / Ohms	R_{off} / Ohms
Mean	2.6	-2.6	1.2×10^5	3.6×10^{10}
Median	2.1	-2.4	5.8×10^4	6.2×10^9
Interquartile Range (IQR)	1.0	2.0	6.0×10^4	2.3×10^{10}
First Quartile (Q1 / 25% percentile)	1.8	-3.5	3.6×10^4	3.5×10^8
Third Quartile (Q3 / 75% percentile)	2.8	-1.5	9.7×10^4	2.3×10^{10}
Lower Whisker	0.3	-6.5	5.9×10^3	8.0×10^6
Higher Whisker	4.3	-0.2	1.9×10^5	5.6×10^{10}

In conclusion, the statistical analysis presented herein, robustly establishes the reproducibility of our graphene-electrode memristor devices across various fabrication runs and device evaluations.

2. Graphene memristor performances in recent studies

Table S3 Graphene-based memristor performances in recent studies

**Device Structure**	**V_{set} / V**	**V_{reset} / V**	**On/Off Ratio**	**Retention / s**	**Reference**
$Gr/TiO_x/AlO_x/Au$	2.6	-2.6	$> 10^5$	$> 6 \times 10^3$	Our work
$Ag/BNO_x/Gr$	0.63	0.1	$< 10^3$	10^4	Ref. S1
$Gr/MoS_{2-x}/Gr$	3	-4	10^4	$> 10^5$	Ref. 15
$Gr/Al_2O_3/MoS_2$	6	-4	$> 10^7$	10^3	Ref. S2
$Gr/MoS_2/SiO_x/Ni$	~2	~-1	$< 10^5$	$\sim 10^3$	Ref. S3
$Gr/AlO_x/TiO_x/ITO$	-2.03	0.87	$> 3 \times 10^2$	10^4	Ref. S4

3. Raman spectrum analysis of as-grown graphene

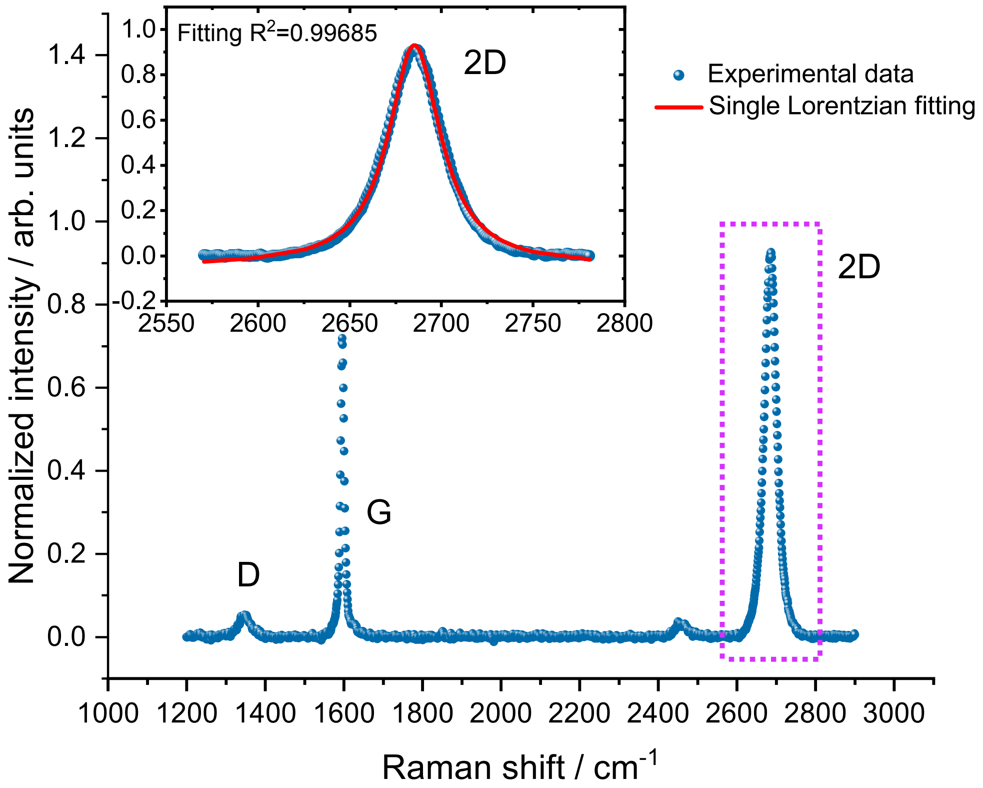

Figure S3. Fitting of as-grown graphene Raman spectrum. Inset: zoom-in of the 2D band spectrum with a single Lorentzian peak fitting (fitting score R^2 = 0.99685).

In our as-grown graphene Raman spectrum (**Figure S3**), I_{2D}/I_G is 3.96 ± 0.16, demonstrating a larger 2D band intensity relative to the G band intensity, and a significant improvement over the graphene from Paragraf reported in our previous work in 2021. Furthermore, the 2D peak is highly symmetric and is well fitted with a single Lorentzian peak with a high fitting score of R^2 = 0.99685. These results confirm the as-grown graphene is monolayer.

Additionally, we present a Raman spectrum typical of more recent batches of as-grown graphene in **Figure S4**. In this spectrum, I_{2D}/I_G is 5.99 ± 0.08 and the 2D peak also displays symmetry with a single Lorentzian fit and a fitting score of R^2 = 0.99392, indicating monolayer graphene. Notably, there is no visible D peak in this spectrum, which suggests a substantially lower density of defects in current batches of as-grown graphene. Table S4 Highlights the steady improvement in the quality of Paragraf's graphene over several years, noting that the memristors presented in this manuscript used graphene produced >1 year ago. It is anticipated that further improvements in memristor performance could be achieved with the recent higher quality graphene.

Table S4. Evolution of the quality of as-grown graphene from Paragraf Ltd. over time.

	I_{2D}/I_G	I_D/I_G
Graphene used as OLED anode[36]	2.25 ± 0.06	0.23 ± 0.02
Graphene used as memristor electrode (this work, **Figures 3** & **S3**)	3.96 ± 0.16	0.25 ± 0.04
Typical recently produced graphene (this work, **Figure S4**)	5.99 ± 0.08	D-peak not visible

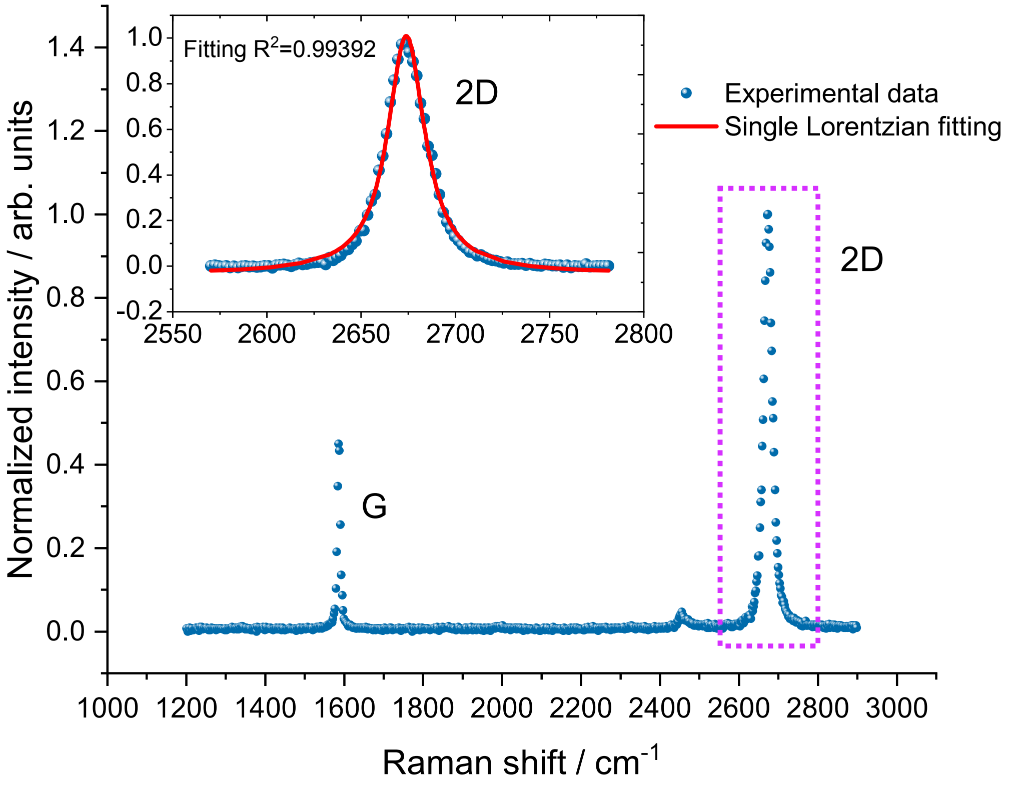

Figure S4. As-grown graphene Raman spectrum from a more recent batch. Inset: zoom-in of the 2D band spectrum with a single Lorentzian peak fitting (fitting score $R^2 = 0.99392$).

Corresponding Authors:

Zhichao Weng: z.weng@qmul.ac.uk

Oliver Fenwick: o.fenwick@qmul.ac.uk

References:

(S1) Zhao, H.; Dong, Z.; Tian, H.; DiMarzi, D.; Han, M.-G.; Zhang, L.; Yan, X.; Liu, F.; Shen, L.; Han, S.-J.; Cronin, S.; Wu, W.; Tice, J.; Guo, J.; Wang, H. Atomically Thin Femtojoule Memristive Device. *Adv. Mater*. **2017**, 29 (47), 1703232. https://doi.org/https://doi.org/10.1002/adma.201703232.

(S2) Vu, Q. A.; Kim, H.; Nguyen, V. L.; Won, U. Y.; Adhikari, S.; Kim, K.; Lee, Y. H.; Yu, W. J. A High-On/Off-Ratio Floating-Gate Memristor Array on a Flexible Substrate via CVD-Grown Large-Area 2D Layer Stacking. *Adv. Mater*. **2017**, 29 (44), 1–7. https://doi.org/10.1002/adma.201703363.

(S3) Krishnaprasad, A.; Dev, D.; Shawkat, M. S.; Martinez-Martinez, R.; Islam, M. M.; Chung, H.-S.; Bae, T.-S.; Jung, Y.; Roy, T. Graphene/MoS2/SiOx Memristive Synapses for Linear Weight Update. *npj 2D Mater. Appl*. **2023**, 7 (1), 22. https://doi.org/10.1038/s41699-023-00388-y.

(S4) Huang, Y.-J.; Lee, S.-C. Graphene/h-BN Heterostructures for Vertical Architecture of RRAM Design. *Sci. Rep*. **2017**, 7 (1), 9679. https://doi.org/10.1038/s41598-017-08939-2.
